# A Printed Dipole Array with Bidirectional Endfire Radiation for Tunnel Communication

**DOI:** 10.3390/s23229137

**Published:** 2023-11-13

**Authors:** Tianfan Xu, Mengchi Xu, Haitao Lu, Xiao Cai

**Affiliations:** Research Center of Applied Electromagnetics, Nanjing University of Information Science and Technology, Nanjing 210044, China; xu18121413148@126.com (T.X.); xumengchi01@163.com (M.X.);

**Keywords:** bidirectional endfire radiation, method of the maximum power transmission efficiency, printed dipole array, tunnel communication

## Abstract

Tunnel communication always suffers from path loss and multipath effects caused by surrounding walls. Meanwhile, the traditional leaky coaxial cables are expensive to deploy, inconvenient to operate, and difficult to maintain, leading to many problems in practical use. To solve the abovementioned problems, a low-profile printed dipole array operating at 3.5 GHz with bidirectional endfire radiation is designed based on the method of maximum power transmission efficiency (MMPTE). By setting two virtual test receiving dipoles at the two opposite endfire directions and then maximizing the power transmission efficiency between the printed dipole array to be designed and the test receiving antennas, the optimal amplitudes and phases for the array elements are obtained. Based on the optimal distributions of excitations, the simulation results show that the proposed eight-element printed dipole array can simultaneously generate two mirrored endfire beams towards opposite directions. Furthermore, the corresponding normalized cross-polarization levels are lower than −22.3 dBi both in the azimuth and elevation planes. The peak endfire gain is 10.7 dBi with maintenance of higher than 10 dBi from 3.23 GHz to 3.66 GHz, which is suitable for tunnel communication.

## 1. Introduction

With the rapid development of modern society, demands for high-quality communication inside coal mines and tunnels are increasing [1,2,3,4,5]. The complex communication environment attenuates the propagation of microwaves, leading to unstable signals or communication interruptions. Considering the special physical structure of the tunnel, popular antenna arrays with a high gain towards the boresight direction are not applicable. Thus, an endfire antenna array with a low profile, high endfire gain, and bidirectional endfire radiation is urgently needed.

Due to the unique advantage of endfire radiation, endfire antennas/antenna arrays have been widely used in 5G terminal [6,7,8,9,10,11,12,13] and millimeter-wave applications [14,15,16,17,18,19,20,21]. Yagi–Uda antenna, known as the most famous endfire antenna and commercial structure, is composed of one driven element, one reflector, and several directors [22,23,24]. Huang first proposed a microstrip array antenna in 1989 [25], which is applied to the recent designs of quasi-Yagi antennas [26,27,28]. Compared to the metal dipole array, microstrip arrays have the advantages of being light weight and low cost. A compact pattern-reconfigurable filtering microstrip quasi-Yagi antenna with a fractional bandwidth of 25% was designed and manufactured in [29]. To achieve a wide bandwidth with a low profile and a compact structure simultaneously, a novel directional microstrip quasi-Yagi antenna array was proposed in [30], whose bandwidth was improved to 17.6%, and the peak gain reached 10 dBi. Furthermore, the microstrip quasi-Yagi antennas cannot realize a real endfire due to the existence of the ground plane, which operates as a reflector.

Unlike the Yagi antenna and various types of quasi-Yagi antenna/antenna arrays, conventional dipole antenna arrays with endfire radiation have wide impedance bandwidths and are easy to manufacture. However, the dipole array size is too large for some applications. A two-dipole endfire antenna with a compact size was presented in [31] by using a meandered line to reduce the spacing while ensuring the endfire performance. Chen proposed a design of a compact wideband endfire filtering antenna in [32], which was smaller in size compared with a classical Yagi–Uda antenna. A four-element compact multibeam endfire dual-polarized antenna array was fabricated in [33], which could generate four beams with two polarizations, covering a wide range of ±41°. Most of the abovementioned antenna arrays are used for endfire radiations in only one direction, which cannot meet the growing requirements in tunnel applications. Thus, a bidirectional endfire radiation antenna array is required for further exploration.

In this study, a low-profile eight-element printed dipole array operating at 3.5 GHz with bidirectional endfire radiation is proposed. A method of the maximum power transmission efficiency for antenna array designs is presented. The corresponding optimization process is briefly summarized in three steps. By setting the two virtual test receiving antennas in the far field and then maximizing the power transmission efficiency between the eight-element array to be designed and the two receiving antennas, the radiated power is concentrated to the desired two mirrored endfire directions. Then, a full-wave simulation is conducted in the electromagnetic simulation software, and the optimized distribution of excitations for the eight elements can be obtained by solving an eigenvalue equation. Fed by the optimal excitations, the proposed eight-element printed dipole array can generate a pair of mirrored endfire beams with an endfire gain of 10.7 dBi, which has potential applications in tunnel communication.

## 2. Method of Maximum Power Transmission Efficiency

The original intention of MMPTE is to optimize the power transmission efficiency between two antennas or array antennas. In the past, the antenna array designs were often aimed at specific antenna performances, such as gain and bandwidth, and were only designed independently as transmitting or receiving. However, in actual wireless communication systems, both the transmitting and receiving terminals exist simultaneously. Therefore, only the power transmission efficiency (PTE) between the transmitting and receiving antennas can be considered the ultimate goal of the antenna array design. Based on this idea, using the PTE between the two antenna arrays as the objective function to optimize the excitation distributions of both the transmitting and receiving arrays is undoubtedly the most effective way to design the antenna array.

**Figure 1:**
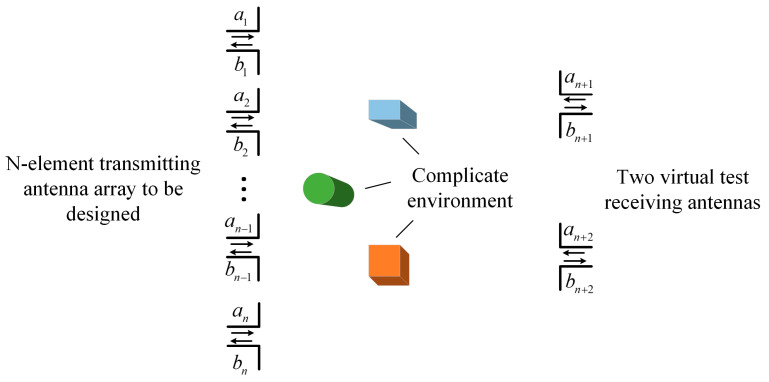
Scheme of the power transmission system consisting of an *n*-element transmitting array to be designed and two virtual test receiving antennas in a complex environment.

As shown in Figure 1, we consider a general wireless communication system with an *n*-element transmitting array to be designed and two virtual test receiving antennas. Then, the entire wireless communication system can be viewed as a (*n* + 2)-port network and then characterized by the scattering parameters, expressed as

(1) $$\left[\begin{array}{l} \left[b_{t}\right]\\ \left[b_{r}\right] \end{array}\right]=\left[\begin{array}{l} \left[S_{tt}]\;[S_{t}{}_{r}\right]\\ \left[S_{rt}]\;[S_{tt}\right] \end{array}\right]\;\left[\begin{array}{l} \left[a_{t}\right]\\ \left[a_{r}\right] \end{array}\right],$$

where the subscript ‘*t*’ and ‘*r*’, respectively, stand for transmitting and receiving and

(2) $$\begin{array}{l} {}[a_{t}]={\left[a_{1},a_{2},\cdot \cdot \cdot ,a_{n}\right]}^{T},\\ {}[a_{r}]={\left[a_{n+1},a_{n+2}\right]}^{T},\\ {}[b_{t}]={\left[b_{1},b_{2},\cdot \cdot \cdot ,b_{n}\right]}^{T},\\ {}[b_{r}]={\left[b_{n+1},b_{n+2}\right]}^{T}. \end{array}$$

denote the normalized incident and reflected waves for the transmitting antenna array and the test receiving antennas, respectively. The power transmission efficiency between the transmitting array and the two test receiving antennas is defined as the ratio of the power delivered to the loads of the receiving antennas to the input power to the transmitting array, expressed as

(3) $$PTE=\frac{\frac{1}{2}({\left|\left[b_{r}\right]\right|}^{2}-{\left|\left[a_{r}\right]\right|}^{2})}{\frac{1}{2}({\left|\left[a_{t}\right]\right|}^{2}-{\left|\left[b_{t}\right]\right|}^{2})}.$$

If the two receiving antennas in the far field are matched, $[a_{r}]=0$ can be achieved, and (3) yields

(4) $$PTE=\frac{\left([A][a_{t}],[a_{t}]\right)}{\left([B][a_{t}],[a_{t}]\right)},$$

where the two matrices [A] and [B] are defined by

(5) $$[A]={\left[S_{rt}\right]}^{H}[S_{rt}],\;[B]=[1]-{\left[S_{tt}\right]}^{H}[S_{tt}].$$

Here, the subscript ‘*H*’ represents the Hermitian operation (conjugate transpose). If the PTE reaches the maximum at $\left[a_{t}\right]$, we have

(6) $$[A][a_{t}]=PTE[B][a_{t}].$$

Furthermore, if the transmitting antenna array elements are well-matched, (6) can be further simplified as

(7) $$[A][a_{t}]=PTE[a_{t}].$$

Equation (7) is an eigenvalue equation, where PTE and $\left[a_{t}\right]$ represent the corresponding eigenvalues and eigenvectors, respectively. Therefore, the maximum value of PTE is the largest eigenvalue of (7), which can be found numerically by using MATLAB. Here, it should be noted that when the number of test receiving antennas is more than one, the eigenvalue equation will have multiple real-number eigenvalues. At this time, the maximum value, which is the maximum transmission efficiency, needs to be selected, and the corresponding eigenvector is the optimal excitation distribution for the antenna array to be designed.

The antenna array design process based on the presented MMPTE can be summarized in the following three steps:

(1) Select the antenna elements and arrange them appropriately to form a transmitting antenna array. Calculate the far-field distance of the transmitting antenna array to be designed according to the practical array arrangement. Introduce one/several test receiving antenna/antennas in the far-field to form a wireless power transmission system.
(2) Choose one of the commercial electromagnetic simulation software, such as Ansoft High-Frequency Structure Simulator (HFSS) or CST studio suite, to conduct a full-wave simulation to obtain the required electromagnetic scattering parameters. In some complex environments, modeling in the software becomes extremely difficult or even infeasible. At this time, the scattering parameters can also be obtained from the measurement by using the multi-port vector network analyzer. In this study, the simulation software HFSS is used, and the scattering matrix is obtained from the full-wave simulation.
(3) Substitute the obtained scattering matrix directly into Equation (7) and obtain the maximum eigenvalue and its corresponding eigenvector. The maximum eigenvalue is the maximum PTE for the wireless power transmission system, and the corresponding eigenvector is the optimal excitation distribution of the transmitting antenna array. Then, a feeding network should be designed to realize the calculated optimal distribution of excitations for the transmitting antenna array. In addition, the optimal distribution of both amplitudes and phases can also be realized by radio frequency circuits, including several phase shifters, attenuators, and power dividers.

The optimization MMPTE can achieve the theoretical maximum value of PTE in wireless power transmission systems when the antenna array arrangement of the structure, the number of antenna elements, and inter-element spacing are determined. While the antenna array arrangement is changed, the maximum PTE is also affected. Furthermore, the optimization process in MMPTE considers the mutual couplings among antenna elements, whereas other design methods and optimization algorithms based on array factors ignore the mutual couplings. Thus, MMPTE provides the possibility and guidance for the design and optimization of small inter-element spacing (less than half-wavelength) antenna arrays.

## 3. Bidirectional Printed Dipole Array Design

### 3.1. Antenna Element Design

**Figure 2:**
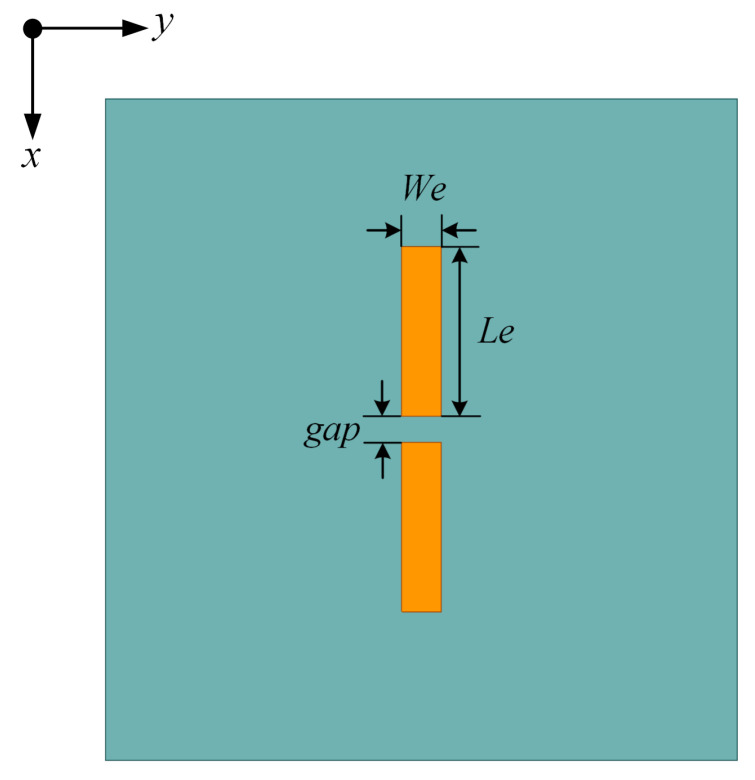
Scheme of the proposed printed dipole.

For tunnel communication, low-profile antennas such as microstrip patch antennas and printed dipoles are preferred because the space left for the whole antenna array is very limited. However, the microstrip patch antenna is not qualified to be an array element because of its extremely low radiated power in the endfire directions. Thus, the printed dipole is selected as the array element due to its benefits of low profile, light weight, small size, low cost, and easy integration. As can be seen in Figure 2, a dielectric printed dipole operating at 3.5 GHz is designed on an FR4 substrate with a thickness of *h*, a relative dielectric constant of 4.4, and a loss tangent of 0.02. The length and width of the two striped arms are *W*_e_ and *L*_e_, respectively. Both the arms are printed on the same side of the substrate. The optimized values for the length and width of the arm and the gap are listed in Table 1. Compared with the traditional metal dipole with a length of $\lambda_{0}/2$ ($\lambda_{0}$ is the wavelength in free space), the length of the proposed printed dipole is only $0.31\lambda_{0}$ due to the presence of the substrate, leading to a more compact antenna size.

**Figure 3:**
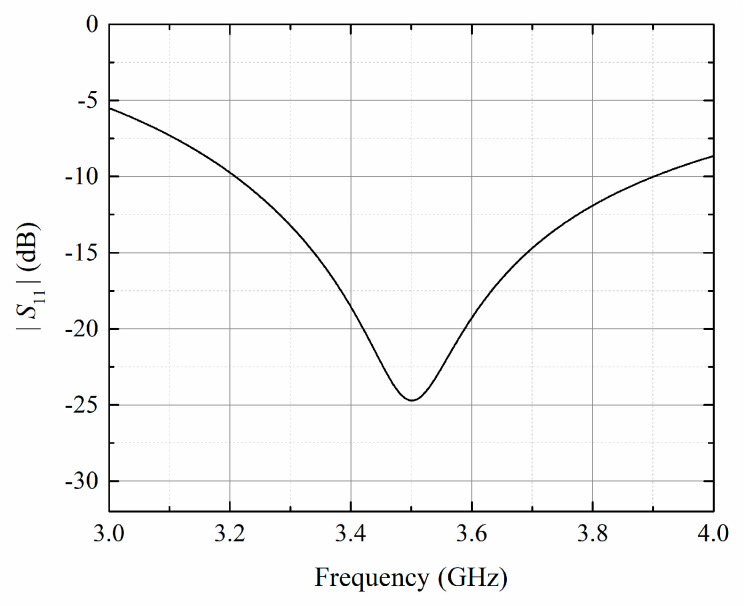
Simulated reflection coefficients of the printed dipole.

The simulated reflection coefficients are shown in Figure 3. It can be seen that the bandwidth of the printed dipole element ranges from 3.21 to 3.90 GHz. The relative bandwidth is calculated to be 19.7%. The reflection coefficient at 3.5 GHz center frequency is below −24.7 dB.

**Figure 4:**
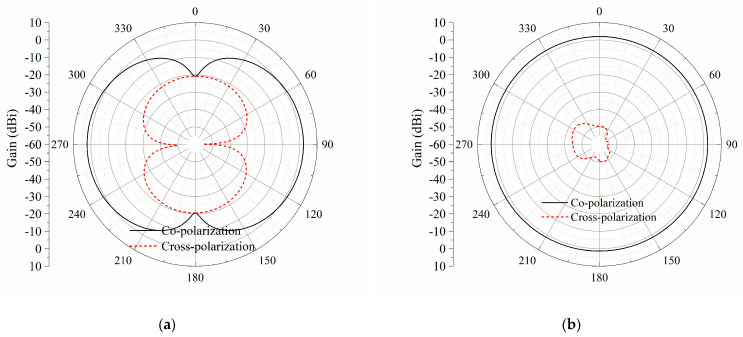
Simulated radiation patterns of the printed dipole. (**a**) *xy*-plane; (**b**) *yz*-plane.

Figure 4 shows the simulated radiation patterns of the two planes for the printed dipole. In the *yz*-plane, the radiation pattern is omnidirectional with an average gain of about 2 dBi. Meanwhile, the corresponding cross-polarization level is extremely low at −55 dB. In the *xy*-plane, the main radiation directions are towards the +*y*-direction and −*y*-direction, sharing the same endfire gain of 2.15 dBi. There is no side lobe, and the gain in ±*x*-directions is −20.7 dBi. The corresponding cross-polarization level is maintained at lower than −22.3 dBi.

### 3.2. Eight-Element Printed Dipole Array Design and Excitation Optimization

**Figure 5:**
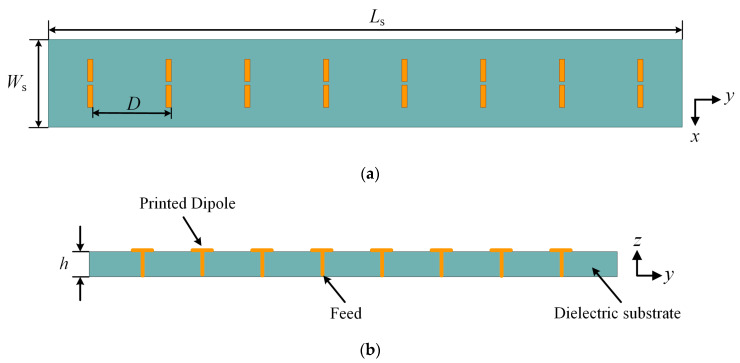
Different views of the printed dipole array. (**a**) Top view; (**b**) side view.

To achieve bidirectional radiation as well as a higher endfire gain, an eight-element printed dipole array is designed and optimized using MMPTE. As is shown in Figure 5, a linear printed dipole array is arranged with a uniform inter-element spacing *D*. The dielectric substrate has a length *L*_s_ of 366.64 mm, a width *W*_s_ of 50.68 mm, and a height *h* of 3 mm. The initial value of *D* is determined to be 45.455 mm (half-wavelength).

**Figure 6:**
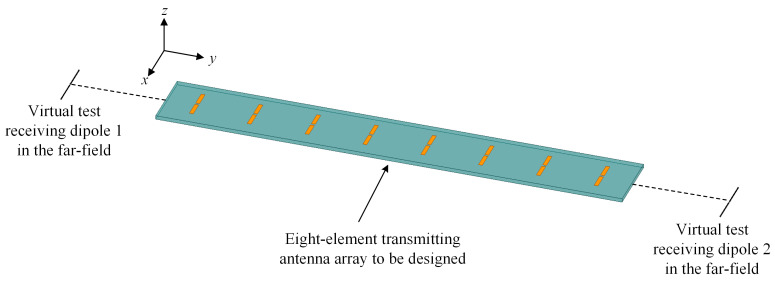
Diagram of the power transmission system including an eight-element transmitting antenna array and two virtual test receiving dipoles.

Once the antenna array arrangement is determined, the far-field distance *r* of the antenna array can be calculated by

(8) $$r\geq \frac{2A^{2}}{\lambda_{0}},$$

where *A* is the antenna array aperture and *λ*_0_ is the wavelength in free space. Then, in order to generate two endfire beams, two test receiving dipoles are set in the ±*y*-directions. It can be seen from Figure 6 that both two test receiving dipoles are in the far-field region, and the spacing between each test receiving dipoles and the antenna array to be designed is the same. Thus, if the PTE between the transmitting antenna and receiving antennas is maximized, the radiated power should be forced to concentrate on the two endfire directions where the two test receiving dipoles are arranged. Hence, the (8 + 2)-port wireless power transmission system is modeled. It should be noted here that the two virtual test receiving dipoles are only used in the optimization process, leading to concentrated power towards the directions in which they are placed.

While the corresponding full-wave simulation is conducted, the simulated scattering matrix is achieved for the following optimization. As a result, the optimal distribution of excitations for the eight array elements is obtained according to (7) and is listed in Table 2.

## 4. Results and Discussion

**Figure 7:**
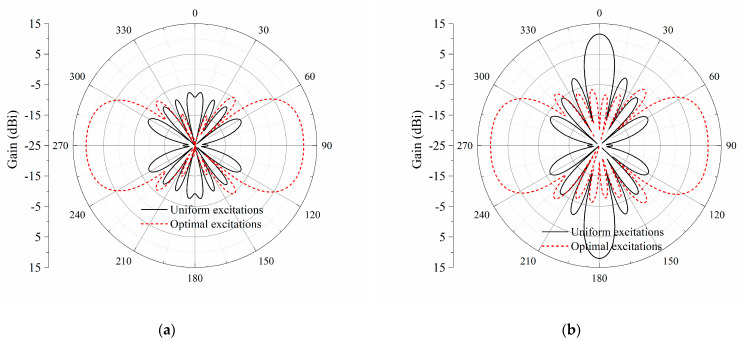
Simulated radiation patterns of the eight-element printed dipole array. (**a**) *xy*-plane; (**b**) *yz*-plane.

**Figure 8:**
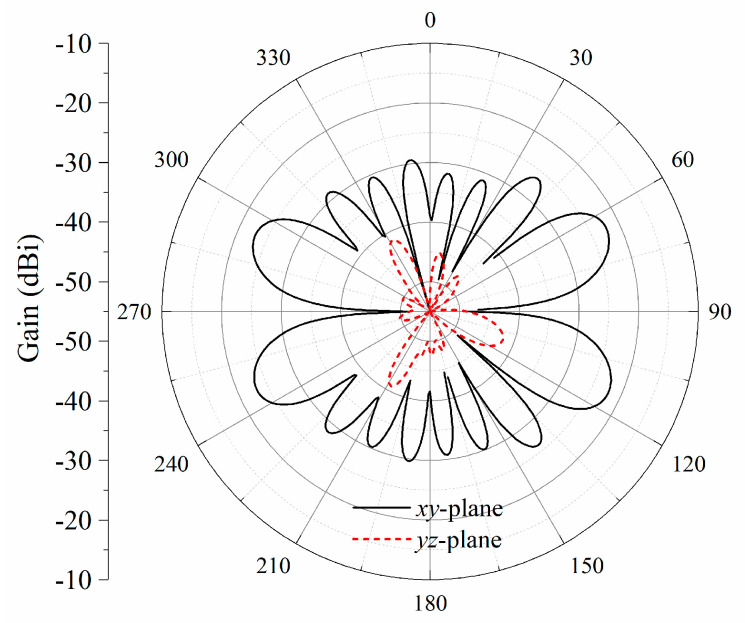
Simulated cross-polarization radiation patterns.

Compared with the uniform distribution of excitations, the radiation patterns of the proposed antenna array fed by optimized excitations are plotted in Figure 7. As shown in Figure 7b, for the antenna array with uniform excitations, a peak gain of 11.5 dBi occurs at ±*z*-directions. Nulls appear at the endfire directions in both the *xy*-plane and *yz*-plane. However, the bidirectional endfire radiation is generated by applying the optimized distributions of amplitudes and phases for the eight elements. The co-polarization endfire gain at ±*y*-directions is the same, i.e., 10.7 dBi, due to the same distance between the test receiving dipole and the printed dipole array. The half-power beam width (HPBW) is about 56°. The side lobe levels for the *xy*-plane and *yz*-plane are 15.8 dB and 12.2 dB, respectively. As shown in Figure 8, the cross-polarization levels in both the *xy*-plane and *yz*-plane are very low, especially in the *xy*-plane.

**Figure 9:**
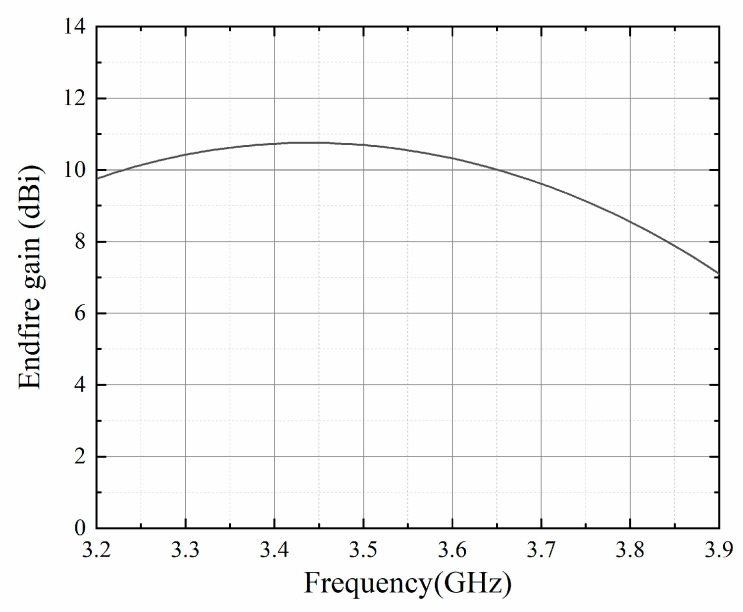
Simulated endfire gain with the changing frequency.

The endfire gain is estimated in Figure 9 within the frequency band from 3.2 GHz to 3.9 GHz, which is also the bandwidth of the printed dipole element. The peak gain of 10.7 dBi is achieved around 3.5 GHz. The endfire gain maintains higher than 10 dBi from 3.23 GHz to 3.66 GHz (430 MHz). When the frequency exceeds 3.66 GHz, the endfire gain decreases faster.

Since the antenna array design based on MMPTE has taken the mutual couplings among antenna elements into consideration, the inter-element spacing could be less than the common half-wavelength. Thus, the antenna array size can be reduced by decreasing the inter-element spacing from λ/2 to 0.4λ or even to λ/3. In practical scenarios, the space for an endfire antenna array is limited. Hence, an antenna array with a compact size is always preferable.

Following the similar design process mentioned in Section 2, the optimized distributions of excitations for different inter-element spacings are listed in Table 3. Here, it should be mentioned that during the optimization, the far-field distance should be recalculated because the antenna array aperture is changed along with the reduction in the inter-element spacing. It can be seen from Table 3 that while the inter-element spacing decreases, the amplitude difference among the array elements increases. Different amplitudes indicate that each element has a different contribution to the synthesized radiation pattern. For the three proposed designs with different inter-element spacings, the amplitude distribution varies from each other, indicating that the main contributing elements are changing. As listed in the middle column of Table 3, the input power delivered to antenna No. 1 and No. 8 is almost sixteen times that of antenna No. 4 and No. 5 in the 0.4λ case.

**Figure 10:**
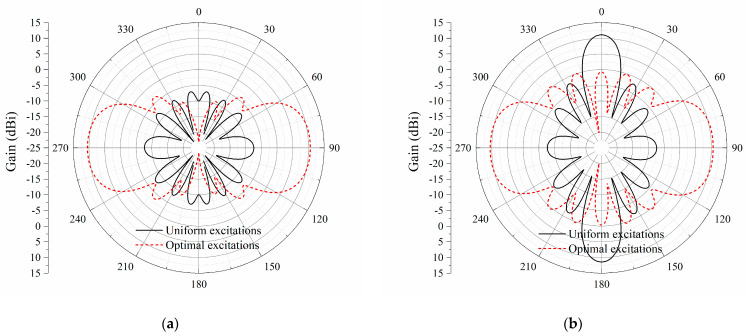
Simulated radiation patterns for the printed dipole array with an inter-element spacing of 0.4*λ*. (**a**) *xy*-plane; (**b**) *yz*-plane.

**Figure 11:**
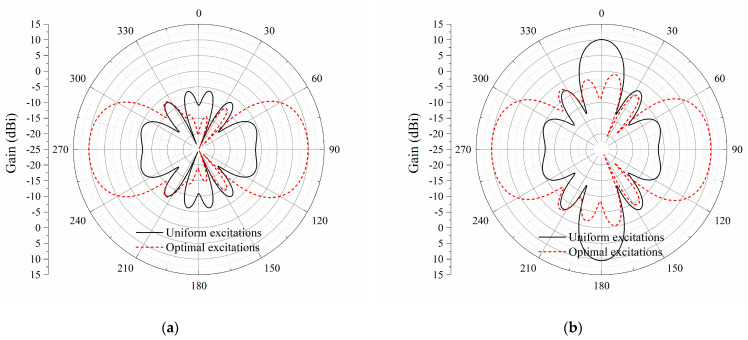
Simulated radiation patterns of the printed dipole array with an inter-element spacing of *λ*/3. (**a**) *xy*-plane; (**b**) *yz*-plane.

By applying the optimized distributions of excitations in the middle and right columns, the radiation patterns for the eight-element printed dipole array with 0.4λ and λ/3 inter-element spacings are plotted in Figure 10 and Figure 11. Although the inter-element spacing and the array size are reduced, the proposed arrays can still realize the bidirectional endfire radiation. As there is always a trade-off between the antenna size and the performance, the endfire gains for the 0.4λ and λ/3 printed dipole arrays are 10.6 dBi and 9.9 dBi, respectively, which is 0.1 dB and 0.8 dB lower than the array with an inter-element spacing of half-wavelength.

**Figure 12:**
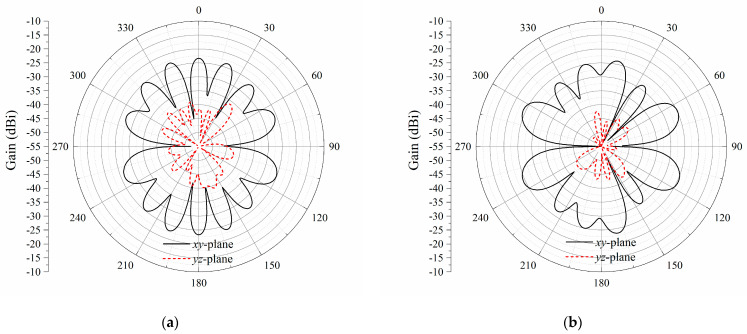
Simulated cross-polarization radiation patterns of different inter-element spacings. (**a**) 0.4*λ*; (**b**) *λ*/3.

Figure 12 shows the simulated cross-polarization radiation patterns for printed dipole arrays with 0.4*λ* and *λ*/3. The cross-polarization levels in the *xy*-plane and *yz*-plane remain very low. The comparisons of the three proposed printed dipole arrays with different inter-element spacings, as well as the publications, are listed in Table 4. By reducing the inter-element spacing to 0.4*λ* and *λ*/3, the overall antenna array size is reduced by 17.3% and 29.5%, respectively. Meanwhile, the cross-polarization gain decreases and the HPBW becomes narrower with a reduction in the inter-element spacing. It should be noted that the change in the endfire gain is only 0.1 dB and 0.8 dB in the 0.4*λ* and *λ*/3 case, respectively, and the benefits of a more compact array size and suppressed cross-polarization level are considerable. Considering the performance, endfire gain, cross polarization, and the realization of bidirectional radiation, the proposed antenna arrays are well-designed and suitable for tunnel communication.

## 5. Conclusions

In this paper, a low-profile eight-element printed dipole array operating at 3.5 GHz with bidirectional endfire radiation is presented based on MMPTE. By placing two virtual test receiving antennas at two mirrored endfire directions, the optimized distribution of amplitudes and phases for the eight elements is obtained. Based on the optimal excitations, the simulation results show that the eight-element printed dipole array can generate a pair of mirrored endfire beams. A peak endfire gain of 10.7 dBi is achieved with maintenance of higher than 10 dBi from 3.23 GHz to 3.66 GHz. The cross-polarization levels are lower than −22.3 dBi both in the azimuth and elevation planes. Furthermore, the performances of the eight-element printed dipole array with small inter-element spacings of 0.4*λ* and *λ*/3 are also analyzed. In addition to the slight 0.1 dB and 0.8 dB reductions in the endifire gain, the reductions in the overall antenna array size are 17.3% and 29.5%, respectively.

In summary, the proposed 3.5 GHz eight-element printed dipole arrays with a low profile and compact size can provide bidirectional endfire radiation with high endfire gain, which is more suitable for the current tunnel communication compared with the traditional coaxial cables or sector beam antennas.

## Figures and Tables

**Table 1:** Parameters of the printed dipole.

Parameter	Value (mm)
*We*	3
*Le*	12.99
*gap*	1.974
*h*	3

**Table 2:** Optimized distribution of excitations.

Antenna No.	Optimized Excitation
1	0.32 V∠−170.5°
2	0.36 V∠1.5°
3	0.41 V∠−180.0°
4	0.36 V∠1.2°
5	0.34 V∠−179.9°
6	0.32 V∠0.7°
7	0.37 V∠−176.3°
8	0.33 V∠0.0°

**Table 3:** Optimized distributions of excitations for different inter-element spacings.

Antenna No.	0.4*λ*	*λ*/3
1	0.48 V∠−1.8°	0.44 V∠−171.4°
2	0.37 V∠−176.8°	0.30 V∠−15.6°
3	0.29 V∠1.3°	0.15 V∠15.6°
4	0.13 V∠−174.3°	0.40 V∠−166.7°
5	0.13 V∠172.0°	0.38 V∠9.0°
6	0.33 V∠−0.6°	0.24 V∠−18.6°
7	0.39 V∠−178.3°	0.40 V∠−172.2°
8	0.49 V∠0°	0.41 V∠0°

**Table 4:** Performance comparisons of the three proposed designs and the publications.

	Method	Type	Frequency (GHz)	Array Size	Endfire Gain (dBi)	Cross- Polarization Gain (dBi)	Bidirectional Endfire Radiation
[5]	Serial-fed	Folded dipole	0.866	100 mm × 29 mm × 1 mm (0.28*λ* × 0.08*λ* × 0.01*λ*)	8.5	<−20	Yes
[13]	Field coupling	AVA array	28	35.3 mm × 36.6 mm × 0.25 mm (3.3*λ* × 3.42*λ* × 0.02*λ*)	13.67	<−14	No
[30]	Mode analysis	Microstrip quasi-Yagi array	5.8	64 mm × 64 mm × 1.5 mm (1.24*λ* × 1.24*λ* × 0.03*λ*)	10.0	<−20	No
[34]	Particle swarm optimization (PSO)	SIW antenna	38	37.8 mm × 21.2 mm × 1.03 mm (4.79*λ* × 2.69*λ* × 0.13*λ*)	5.0	<−15	No
[35]	Artificial bee colony (ABC) algorithm	ELPD array	5.8	170 mm × 80 mm × 7.51 mm (3.29*λ* × 1.55*λ* × 0.01*λ*)	10.1	Not Given	No
[36]	Decoupling network	Super-directive array	1.0	Not Given -	8.14	Not Given	No
[37]	Series-fed	Traveling wave array	0.425	190 mm × 190 mm × 562.5 mm (0.25*λ* × 0.25*λ* × 0.75*λ*)	8.63	Not Given	No
This work (*λ*/2)	MMPTE	Printed dipole	3.5	345.8 mm × 47.56 mm × 3 mm (4.04*λ* × 0.54*λ* × 0.04*λ*)	10.7	<−22.3	Yes
This work (0.4*λ*)	MMPTE	Printed dipole	3.5	285.8 mm × 47.56 mm × 3 mm (3.33*λ* × 0.54*λ* × 0.04*λ*)	10.6	<−23.0	Yes
This work (*λ*/3)	MMPTE	Printed dipole	3.5	243.8 mm × 47.56 mm × 3 mm (2.84*λ* × 0.54*λ* × 0.04*λ*)	9.9	<−23.3	Yes

## Data Availability

Data are contained within the article.
